# The influence of organizational caring on mobile phone addiction in undergraduate nursing students: The chain mediating role of perceived stress and self-control

**DOI:** 10.1016/j.heliyon.2024.e37679

**Published:** 2024-09-10

**Authors:** Wenkai Zheng, Wenjin Chen, Jiao Fang, Na Li, Junchao Huang, Leilei Wang, Meifang Wang, Xiujuan Feng, Chunni Heng, Yunlong Tan

**Affiliations:** a School of Basic Medicine, Inner Mongolia Medical University, Hohhot, 010110, China; b Peking University HuiLongGuan Clinical Medical School, Beijing, 100096, China; c College of Nursing and Rehabilitation, Xi'an Jiaotong University City College, Xi’an, 710018, China; d Department of Endocrinology, Tangdu Hospital, The Fourth Military Medical University, Xi’an, 710038, China

**Keywords:** Organizational caring, Nursing students, Perceived stress, Self-control, Mobile phone addiction

## Abstract

**Background:**

The incidence of mobile phone addiction (MPA) is increasing in undergraduates and may lead to depression, anxiety, and sleep disorders. Undergraduate nursing students are important group for clinical services; therefore poor mental health has an important implications for the quality of future nursing work and the relationship between nurses and patients.

**Objectives:**

To explore the connection between organizational caring and MPA in Chinese undergraduate nursing students and to investigate the mechanisms of perceived stress and self-control in this relationship by constructing a chain mediation model.

**Design:**

A cross-sectional study.

**Methods:**

A total of 900 participants (age range 18–25 years, *M* = 20.99, *SD* = 1.58, 94.0 % female) were recruited from 10 universities in China. Data were collected using an online survey between February and March 2023. Organizational caring, subjective stress, self-control ability, and MPA among undergraduate nursing students were assessed.

**Results:**

(1) The prevalence of MPA was 34.9 % (314/900). (2) MPA in undergraduate nursing students was negatively correlated with self-control (*r* = −0.468, *p* < 0.001) and organizational caring (*r* = −0.156, *p* < 0.001), and positively correlated with perceived stress (*r* = 0.362, *p* < 0.001). (3) Perceived stress and self-control mediated the relationship between organizational caring and MPA, and the relative mediating effect values were 16.6 % and 17.2 %, respectively. (4) Organizational caring had an indirect effect on MPA through the chain mediation effect of perceived stress and self-control, and the relative mediating effect value was 19.1 %.

**Conclusion:**

Organizational caring, perceived stress and self-control directly influenced MPA among undergraduate nursing students. Additionally, organizational caring indirectly affected MPA through perceived stress and self-control. To further mitigate MPA among students, nursing managers and educators should enhance organizational caring, reduce perceived stress, and improve self-control abilities.

## 1 Introduction

Mobile phone addiction (MPA) refers to a new type of behavioral addiction in which individuals frequently and excessively use their mobile phone and are unable to control such use behavior through autonomous consciousness [1]. An epidemiological survey reported that the prevalence of MPA between 19.0 % and 72.0 % among undergraduate nursing students [2]. Compared to other countries, the prevalence of MPA among undergraduate nursing students in China is notably higher [[2], [3], [4], [5], [6]]. Medical students often face greater academic pressure than students in other degree programs, and studies have shown an association between high academic pressure and MPA [7]. Nursing students, who experience significant academic stress, are at an elevated risk of developing MPA [6]. Nursing students constitute a vital reserve force for clinical nursing work. As the primary providers of medical services, nurses' mental health status is directly linked to the quality of nursing care and the nurse–patient relationship. Therefore, focusing on nursing students’ mental health is important.

MPA impairs individuals’ daily psychosocial function of individuals and, in severe cases, can lead to problems such as depression, anxiety, loneliness, inattention, cognitive impairment, decreased immunity, and sleep disorders [2,3,8,9]. Therefore, the factors that influence MPA are important to identify. Domestic and foreign scholars have actively discussed the influencing factors of MPA, finding that the degree of organizational caring individuals perceive is a protective factor against MPA [10,11]. However, how organizational caring affects the internal mechanisms of MPA remains unclear. Therefore, this study aims to explore the direct and indirect effects of organizational caring on MPA and provide new insights for designing strategies to mitigate MPA among undergraduate nursing students.

### 1.1 Effects of organizational caring on mobile phone addiction

Organizational caring refers to individuals' perceptions of love, concern, and care received from others in their external environment, which in turn influences the cognition and behavior of organizational members [12]. Organizational caring may influence nursing undergraduates’ caregiving abilities and professional burnout [13]. Research has found that the lack of organizational caring is an objective factor hindering undergraduate nursing students from providing care to patients [14]. Therefore, for undergraduate nursing students, it may be more important to focus on how organizational caring during their campus period affects their behavior and psychological state. The stress they experience during university results not only from adjusting to the environment, completing their studies, and communicating with others, but also from changing roles from nursing students to clinical nurses. Qin et al. proposed that stress could cause behavioral problems, with MPA being a typical example [15]. When individuals perceive more care from the outside world, they have more internal psychological resources and are better able to cope with stress [16]. Thus, good social support can alleviate the stress of daily life and learning, contributing to positive physical and mental health. Organizational caring is part of social support, and both have significant positive effects on individuals [17]. Previous research has consistently shown a negative association between social support and MPA [18]. This finding is consistent with the social support buffer hypothesis and compensatory internet use theory [19,20], which suggest that individuals who lack social support will spend more time seeking it through mobile phone use, thereby developing MPA.

### 1.2 Mediating role of perceived stress

Perceived stress refers to the physical and mental tension and discomfort that individuals experience when perceiving stimulating events and threats [21]. High perceived stress is positively correlated with anxiety, depression, and addictive behaviors [[22], [23], [24]]. Research has found a positive correlation between anxiety, depression, and addictive behaviors [23,25,26]. General strain theory [27] suggests that negative experiences resulting from stress or tension can contribute to the emergence of various behavioral problems. Within this framework, MPA can be understood as a coping strategy adopted by students to alleviate stress and tension. Organizational caring has a robust negative predictive effect on individual perceived stress levels [28]. Guided by the main effect model of social support [29], it is apparent that social support has significant implications for mental health, as it influences individuals’ cognitive evaluations of stress, mitigates adverse reactions, and fosters adaptive coping responses. When individuals perceive high levels of social support, they tend to exhibit greater self-control, reduce impulsive behaviors, and maintain rational thoughts to align their behavior more with public expectations when facing stressful events. Social support plays a crucial role in mitigating the adverse effects of negative events on the physical and mental well-being of individuals [30].

### 1.3 Mediating role of self-control

Self-control refers to an individual's internal ability to resist external temptations and effectively achieve their personal goals [31]. Previous research has consistently demonstrated that self-control plays a protective role in the development of MPA [25]. The emergence of addictive behaviors is intricately linked to the presence of diminished self-control and inadequate self-regulation abilities among individuals [25]. Individuals with lower ability of self-control are at a higher risk of developing MPA. In daily life, individuals inevitably encounter stressful events and require self-control resources to complete tasks. However, self-control resources are limited, and when exhausted, subsequent task performance decreases, resulting in self-control failure. Organizational caring, as a social resource can be used to supplement self-control resources, which is consistent with the views of Chen et al. [32]. According to self-control resources theory [33], abundant energy resources can promote self-control, and care from parents, classmates, and teachers can significantly increase individuals' energy resources, improving their self-control ability.

### 1.4 Chain mediating role of perceived stress and self-control

Studies have consistently shown a significant predictive negative relationship between organizational caring and perceived stress [28], with perceived stress displaying a negative correlation with self-control [15]. Perceived stress reduces self-control resources in two ways. First, perceived stress creates negative emotions, which require individuals to consume self-control resources in order to adjust to them. Second, perceived stress triggers undesirable cognitive activities such as rumination, which requires self-control to inhibit [[34], [35], [36]]. Self-control has been identified as a negative predictor of the propensity for MPA [25]. According to the use-satisfaction theory [37], mobile phones, as a mass medium, have the capacity to provide satisfaction and happiness, effectively alleviating the negative emotions resulting from pressure experienced by university students. However, excessive reliance on this psychological experience without proper behavioral control can lead to excessive mobile phone usage, eventually culminating in mobile phone dependency or addiction.

### 1.5 Study purpose

**Fig. 1:**
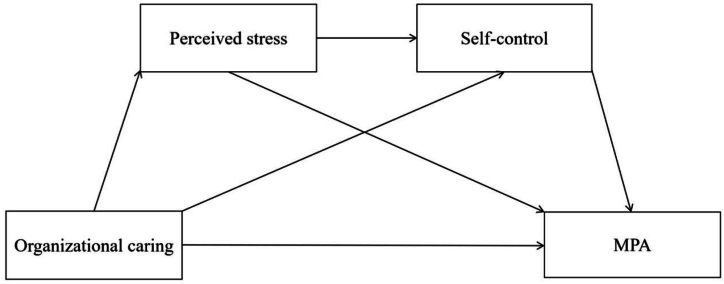
Hypothetical conceptual model.

Building upon the aforementioned analysis, the primary objective of this study is to investigate the correlation between organizational caring, perceived stress, self-control, and MPA among undergraduate nursing students. Additionally, it aims to elucidate the specific pathway through which organizational caring impacts MPA among undergraduate nursing students, with the ultimate goal of offering novel perspectives for nursing educators and managers to ameliorate MPA in this population. Expanding upon prior studies, this research formulates the subsequent hypotheses: (1) A negative association exists between organizational caring and MPA. (2) The relationship between organizational care and MPA may be mediated by perceived stress. (3) The relationship between organizational caring and MPA may be mediated by self-control. (4) Organizational caring may impact MPA among undergraduate nursing students by means of the chain mediation of perceived stress and self-control. The hypothetical conceptual model for this study is illustrated in Fig. 1.

## 2 Method

### 2.1 Design and participants

A convenience sampling method was used. From February to March 2023, nursing students from 10 universities in Shaanxi Province, China, were selected as the survey subjects. Inclusion criteria were the following: (1) four-year undergraduate nursing students; (2) nursing students who voluntarily gave their informed consent. Exclusion criteria were the following: (1) nursing students who were not in school for various reasons, such as being suspended or joining the army. The sample size for this study was calculated using formula N = Z^2^ × [P × (1-P)]/E ^2^, where N represents the required sample size, Z corresponds to the statistic, P signifies the probability value, and E denotes the desired error value. For this study, a 95 % confidence interval was utilized, resulting in Z = 1.96, while an error margin of 4.0 % was deemed acceptable. By applying these parameters, the sample size necessary for this study was estimated. The prevalence of MPA among medical students has been found to be approximately 41.9 % in previous studies [38]. To ensure the statistical reliability and validity of our findings, a minimum sample size of 584 participants was determined to be necessary for this study.

### 2.2 Procedure

The investigators contacted the person in charge of each university to provide a comprehensive explanation of the study's objectives and content, and after obtaining consent, the researchers sent pre-designed questionnaires to the contact person's WeChat to be forwarded to undergraduate nursing students. The questionnaires were collected by the training staff and administered using the Questionnaire Star platform to ensure rigorous quality control. Quality control was as follows: informing participants of the anonymity of their responses and their ability to withdraw from the study at any time; the questionnaire incorporated mandatory and logically structured response options; to prevent duplicate responses, each IP address was limited to a single submission; incentives were offered, including small gifts for participants who completed the questionnaire, as a token of appreciation. A total of 913 questionnaires were collected for this study. Of these, 13 questionnaires were excluded: 4 due to missing responses, 3 due to high response homogeneity across all items, and 6 for having completion times of less than 3 min. Consequently, 900 valid questionnaires were retained for data analysis.

### 2.3 Measurements

#### 2.3.1 Demographic characteristics

A demographics questionnaire was utilized to gather information regarding participants’ characteristics, encompassing variables such as age, gender, grade, only child in the family, type of residence, and family finances.

#### 2.3.2 Organizational caring

Organizational caring was assessed using the Organizational Climate for Caring Questionnaire (OCCQ) [39,40], which included 30 items along three dimensions of organizational caring. The OCCQ is scored on a range of 30–180, with higher scores indicating a stronger perception of organizational caring. In previous studies, the OCCQ has demonstrated robust reliability and validity, with a reported Cronbach's alpha coefficient of 0.93 [40]. In the current study, the Cronbach's alpha coefficient was calculated to be 0.97, indicating excellent reliability.

#### 2.3.3 Perceived stress

The perceived stress levels were measured by the Perceived Stress Scale (PSS) [41], which included 14 items along two dimensions. The PSS is scored on a range of 14–70, with higher scores indicating elevated levels of perceived stress. Previous research has consistently demonstrated the PSS's strong reliability and validity, with a reported Cronbach's alpha coefficient of 0.79 [42]. In the current study, the calculated Cronbach's alpha coefficient was 0.74, indicating satisfactory reliability.

#### 2.3.4 Self-control

Self-control ability was measured by the Self-control Scale (SCS) [31], which included 19 items along five dimensions. The SCS is scored on a range of 19–95, with higher scores indicating higher levels of self-control. Previous research has consistently established the strong reliability and validity of the SCS, reporting a Cronbach's alpha coefficient of 0.84 [43]. In the current study, the calculated Cronbach's alpha coefficient for the SCS was 0.88, indicating good reliability.

#### 2.3.5 Mobile phone addiction

MPA was measured by the Mobile Phone Addiction Tendency Scale (MPATS) [44], which included 16 items along four dimensions. The MPATS is scored on a range of 16–80, with scores ≥48 indicating MPA. The higher the score, the higher the degree of MPA [44]. Previous studies have established the MPATS's strong reliability and validity, reporting a Cronbach's alpha coefficient of 0.88 [45]. In the present study, the Cronbach's alpha coefficient was calculated to be 0.93, indicating good reliability.

### 2.4 Statistical analysis

Statistical analyses were performed using SPSS 27.0. The Gaussian distribution of the data was assessed using the Kolmogorov-Smirnov (K-S) single-sample test and P-P plot. The data were normally distributed. First, Hartman's single-factor test was used to check for common method bias [46]. Second, categorical variables were presented as frequency and percentage. Continuous variables were reported as mean and standard deviation. Chi-square tests and independent samples *t*-test were used to compare demographic data and other variable (organizational caring, perceived stress, and self-control) between the MPA and N-MPA groups. Third, Pearson's correlation analysis was used to explore the correlations among organizational caring, perceived stress, self-control, and MPA. Finally, Model 6 of the SPSS PROCESS macro, developed by Hayes, was employed to investigate the chain mediating roles of perceived stress and self-control between organizational caring and MPA [47]. This method is based on ordinary least squares regression and bootstrapping. Furthermore, to assess the impact of organizational caring on MPA, a bias–corrected percentile bootstrap confidence interval with 95 % coverage was calculated using 5,000 bootstrap samples. Age, gender, grade, one-child status, type of residence, and family finances were included as covariates in the model. A value of *p* < 0.05 was considered statistically significant.

## 3 Results

### 3.1 Common method bias tests

The exploratory factor analysis extracted 12 factors with eigenvalues greater than 1, and the first common factor explained 24.3 % of the variance, which is less than 40.0 %. Thus, this study was considered to have no serious common method bias.

### 3.2 Sample characteristics

Table 1 presents an overview of the sample characteristics. The mean age of the 900 undergraduate nursing students was 20.99 (SD = 1.58) years, with an age ranging from 18 to 25 years. Of these participants, 94.0 % were female, 78.3 % were from families with more than one child, 54.5 % hailed from rural areas, and 82.1 % rated their family's economic status as middle class. The distribution of the students was 21.7 %, 22.9 %, 28.9 %, and 26.5 % for the four grades, respectively.

**Table 1:** Sample characteristics (N = 900).

Variables	N (%)	Mean (SD)
Age (years)		20.99 (1.58)
Gender		
Male	54 (6.0)	
Female	846 (94.0)	
Grade		
Freshmen	195 (21.7)	
Sophomores	206 (22.9)	
Juniors	260 (28.9)	
Senior	239 (26.5)	
Only child in the family		
Yes	195 (21.7)	
No	705 (78.3)	
Type of Residence		
Downtown	206 (22.9)	
Suburb	203 (22.6)	
Rural areas	491 (54.5)	
Family finances		
Low income	142 (15.8)	
Middle income	739 (82.1)	
High income	19 (2.1)	

### 3.3 Descriptive statistics and comparisons

As shown in Table 2, 314 of the 900 participants met the threshold for MPA (34.9 %). Juniors exhibited a higher prevalence of MPA compared to students in other grades (*p* = 0.024). No statistically significant differences were found between the two groups in terms of age (*p* = 0.243), **gender (*****p*****= 0.861)**, one-child (*p* = 0.861), type of residence (*p* = 0.219), and family finances (*p* = 0.280). The MPA group exhibited significantly higher levels of perceived stress and lower levels of organizational caring and self-control compared to the control group. (all *p* < 0.001).

**Table 2:** Comparison of demographic characteristics and variables among the two groups.

Variables	N - MPA (N = 586)	MPA (N = 314)	*χ*^*2*^/*t*	*p*
Age	21.03 ± 1.55	20.90 ± 1.65	1.169	0.243
Gender			0.117	0.733
Male	34 (63.0 %)	20 (37.0 %)		
Female	552 (65.2 %)	294 (34.8 %)		
Grade			9.479	0.024
Freshmen	120 (61.5 %)	75 (38.5 %)		
Sophomores	134 (65.0 %)	72 (35.0 %)		
Juniors	158 (60.8 %)	102 (39.2 %)		
Senior	174 (72.8 %)	65 (27.2 %)		
One-child			0.031	0.861
Yes	128 (65.6 %)	67 (34.4 %)		
No	458 (65.0 %)	247 (35.0 %)		
Type of Residence			3.038	0.219
Downtown	135 (65.5 %)	71 (34.5 %)		
Suburb	122 (60.1 %)	81 (39.9 %)		
Rural areas	329 (67.0 %)	162 (33.0 %)		
Family finances			2.547	0.280
Low income	97 (68.3 %)	45 (31.7 %)		
Middle income	474 (64.1 %)	265 (35.9 %)		
High income	15 (78.9 %)	4 (21.1 %)		
Organizational Caring	131.13 ± 27.45	122.55 ± 25.88	4.556	＜0.001
Perceived stress	38.81 ± 6.01	42.43 ± 4.90	−9.176	＜0.001
Self-control	64.15 ± 10.24	57.15 ± 7.54	10.665	＜0.001

Note: N - MPA = Non - Mobile Phone Addiction; MPA = Mobile Phone Addiction.

### 3.4 Correlation between variables

In this study, MPA among undergraduate nursing students were a significantly negatively correlated with organizational caring (*r* = −0.156, *p* < 0.001) and self-control (*r* = −0.468, *p* < 0.001), and significantly positively correlated with perceived stress (*r* = 0.362, *p* < 0.001). The results are shown in Table 3.

**Table 3:** Analysis of related variables.

Variables	1	2	3	4
1. Organizational caring	1			
2. Perceived stress	−0.162***	1		
3. Self-control	0.152***	−0.515***	1	
4. MPA	−0.156***	0.362***	−0.468***	1
Mean	128.14	40.07	61.71	41.46
Standard deviation	27.21	5.90	9.96	10.12

Note: MPA = Mobile Phone Addiction.

***p < 0.001.

### 3.5 Mediation analysis of perceived stress and self-control between organizational caring and mobile phone addiction

We examined the mediating roles of perceived stress and self-control in the relationship between organizational caring and MPA. The age, gender, grade, one-child status, type of residence, and family finances were controlled for, and the findings are presented in Table 4. According to the results of the regression analysis, it was found that organizational caring negatively predicted perceived stress (*β* = −0.161, *p* < 0.001) and MPA (*β* = −0.074, *p* < 0.05), while positively predicting self-control (*β* = 0.072, *p* < 0.05). Perceived stress was found to negatively predict self-control (*β* = −0.504, *p* < 0.001) and positively predict MPA (*β* = 0.163, *p* < 0.001). Furthermore, self-control was found to have a negative predictive effect on MPA (*β* = −0.374, *p* < 0.001).

**Table 4:** Mediating effect of perceived stress and self-control in the relationship between organizational caring and mobile phone addiction.

Regression model		Overall fit index			Significance of regression coefficients	
Dependent variable	Independent variable	*R*	*R*^*2*^	*F*	*β*	*t*
Perceived stress		0.203	0.041	5.463		
	Organizational caring				−0.161	−4.894***
Self-control		0.530	0.281	43.449		
	Perceived stress				−0.504	−17.360***
	Organizational caring				0.072	2.484*
MPA		0.498	0.248	32.576		
	Perceived stress				0.163	4.754***
	Self-control				−0.374	−10.906***
	Organizational caring				−0.074	−2.478*

Note: MPA = Mobile Phone Addiction.

*p < 0.05, **p < 0.01, ***p < 0.001.

**Fig. 2:**
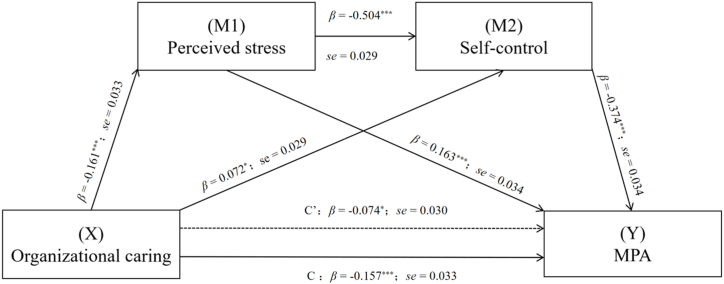
The chain mediation of perceived stress and self-control in the relationship between organizational caring and MPA with standardized beta values and standard error. Note: *p < 0.05, **p < 0.01, ***p < 0.001.

The mediation analysis revealed a significant chain mediation effect of perceived stress and self-control in the relationship between organizational caring and MPA. The chain mediation effect was found to be −0.083. Specifically, the mediation effect was observed through three mediation chains: (1) organizational caring → perceived stress → MPA (effect value: 0.026, 95 % CI: 0.047 to −0.010), (2) organizational caring → self-control → MPA (effect value: 0.027, 95 % CI: 0.055 to −0.003), and (3) organizational caring → perceived stress → self-control → MPA (effect value: 0.030, 95 % CI: 0.048 to −0.016). The results are shown in Table 5 and Fig. 2.

**Table 5:** Total, direct, and indirect effects of organizational caring on mobile phone addiction.

Paths	Effect	Boot SE	Bootstrap 95 % (CI)		Relative Mediation Effect
			BootLL CI	BootUL CI	
Total effect of X on Y	−0.157	0.033	−0.222	−0.092	
Direct effect of X on Y	−0.074	0.030	−0.132	−0.015	47.1 %
Indirect effects 1 (X →M1→Y)	−0.026	0.009	−0.047	−0.010	16.6 %
Indirect effects 2 (X →M2→Y)	−0.027	0.013	−0.055	−0.003	17.2 %
Indirect effects 3 (X →M1→M2→Y)	−0.030	0.008	−0.048	−0.016	19.1 %
Total indirect effect of X on Y	−0.083	0.021	−0.127	−0.046	52.9 %

Note：*N* = 900. Number of bootstrap samples for Bias-corrected bootstrap confidence intervals: 5,000. Level of confidence for all confidence intervals: 95 %. X = Organizational Caring; M1 = Perceived stress; M2 = Self-control; Y = MPA.

## 4 Discussion

The findings of this study show that: (1) MPA among undergraduate nursing students exhibits a negative correlation with organizational caring and self-control, while showing a positive correlation with perceived stress; (2) perceived stress plays a mediating role in organizational caring and MPA; (3) self-control plays a mediating role in the relationship between organizational caring and MPA; and (4) perceived stress and self-control collectively exert a chain-mediated effect on the relationship between organizational caring and MPA among undergraduate nursing students.

### 4.1 Effect of organizational caring on mobile phone addiction

Organizational caring had a protective effect for MPA among undergraduate nursing students. Students who perceived more organizational caring were less likely to develop MPA than those who perceived less or no organizational caring. This study is similar with previous findings from studies conducted with adolescents and other groups [20,48,49]. This finding is in line the social support hypothesis and compensatory Internet use theory [19,20], which can be used to explain why organizational caring reduces MPA among undergraduate nursing students. Students who perceive more organizational caring have richer psychological resources, and when faced with pressure arising from learning and social activities, they will actively seek the help of teachers or peers actively mobilizing psychological and social resources to cope, decreasing the use of mobile phones as a way to release pressure. Enhancing the level of care provided to undergraduate nursing students by teachers and other important personnel is conducive to reducing their MPA.

### 4.2 Mediation through perceived stress

The present study revealed that perceived stress mediates the relationship between organizational caring and MPA. Organizational caring negatively predicted perceived stress; the higher the perceived organizational caring, the lower the perceived stress, which is consistent with previous studies [28,29]. When students are under pressure, care from teachers and classmates can increase their supportive resources, thereby weakening the influence of pressure. For undergraduate nursing students, organizational caring plays an important role in improving their psychological defense mechanisms, reducing the adverse effects of stressful events, and improving their ability to cope with stress, which can make them more successful in their future nursing careers. The findings of this study indicated a positive correlation between perceived stress and MPA among undergraduate nursing students, confirming the general stress theory that stress leads to behavioral problems. The higher the degree of perceived stress, the easier it is for students to reduce stress through problem behaviors (such as indulging in games). When focusing on the effects of perceived stress on MPA, greater attention should be paid to protective factors against perceived stress, including organizational caring.

### 4.3 Mediation through self-control

This study showed a mediating effect of self-control between organizational caring and MPA. Organizational caring can positively predict self-control; the more organizational caring individuals perceive, the better their self-control. This finding aligns with previous research investigating the mediating effect of self-control in the relationship between social support and problem behaviors [32]. This is likely because organizational caring, as an important social resource, plays a role in promoting physical and mental well-being. It can provide individuals with a sense of security sufficient for them to feel recognized and accepted by others, which is conducive to establishing a good self-image, acquiring self-esteem, and improving self-efficacy, resulting in strong self-control [50]. When faced with various stressors, individuals with stronger self-control are better able to regulate and monitor their emotions, cognition, and behaviors; think about problems from multiple perspectives; and deal with problems more appropriately way; thus they exhibit fewer problematic behaviors, such as MPA [15].

Moreover, according to the self-control resource model, individuals' self-control behavior is related to their own self-control resources, which are consumed when generating self-control and control behavior. When self-control resources are insufficient, self-control behaviors fail and people will be prone to problematic behaviors [33]. If the resources consumed by self-control behaviors can be replaced in time, individuals can engage in positive cognitive behaviors in response to negative stressors and reduce problematic behaviors. Organizational caring can reduce individuals’ MPA by improving their self-control. This also suggests that the MPA can be reduced by improving organizational caring and self-control.

### 4.4 Mediation through perceived stress and self-control

Our study revealed that organizational caring indirectly affects the MPA of undergraduate nursing students through the chain mediation of perceived stress and self-control. Prior research has consistently shown that perceived stress is a negative predictor of self-control [15,[34], [35], [36]]. When experiencing low perceived stress, individuals may need to consume fewer self-control resources, while high perceived stress can significantly deplete these resources. This can be attributed to the finite self-control theory, which suggests that individuals possess limited self-control resources within a certain time frame. Under high-pressure circumstances, the consumption of these resources intensifies, leading to a decline in self-control. In general, the higher the degree of organizational caring undergraduate nursing students perceive, the richer the supportive social resources, the lower the perceived pressure in the learning process and social activities, the fewer self-control resources are consumed, the less easily they can engage in problematic behaviors, to escape, and the more reasonably they can make a choice to control themselves from mobile phone overuse, thereby reducing the risk of MPA.

This study had several limitations that should be acknowledged. First, in this study, the participant consisted of 94.0 % females and 6.0 % males. Future research should consider incorporating a higher proportion of male participants to achieve more comprehensive and representative findings. Second, this study used self-assessment questionnaires, which may induce recall and self-report bias. Since individuals have different willingness and understanding ability to fill in the scale, coupled with the strong subjectivity of the self-assessment questionnaires, which affects the authenticity of the answers and research results, may cause some errors. Although our data were professionally assessed by the researchers and tested for common methodological biases, the accuracy of the findings may still be reduced. Future research should include other methodological approaches. Third, the relationship between organizational caring and MPA is multifaceted. This study only focused on examining the mediating effect of perceived stress and self-control, without considering other influential factors. Future research should incorporate additional relevant variables to construct a more comprehensive and systematic model. Fourth, while the application of mediation analysis in cross-sectional studies has inherent methodological limitations, this study provides preliminary insights into the association between organizational caring and MPA, validating theoretical hypotheses. Future research can further investigate this relationship through longitudinal studies for a more robust understanding.

## 5 Conclusions

The study found that, among undergraduate nursing students, organizational caring not only directly negatively predicts MPA, but also indirectly influences MPA through the chain mediation of perceived stress and self-control. This means that more organizational caring leads to lower perceived stress among undergraduate nursing students, thereby enhancing self-control abilities and reducing the risk of MPA.

In conclusion, this study enriches the theoretical research on MPA and elucidates the mechanism through which organizational caring influences MPA. This also provides a new direction for future efforts to improve MPA among undergraduate nursing students.

## Funding

This research was supported by the 10.13039/501100012166National Key Research and Development Program of China (2021YFC2501504).

## Data availability statement

No research-related data are stored in publicly available repositories, and the data will be made available on request.

## Ethics statement

The study was reviewed and approved by the Ethics Committee of Tangdu Hospital (approval number: TDLL–202311–17). The attributes, benefits, uses, and disadvantageous effects of the study were explained to all participants and informed consent was also obtained.

## CRediT authorship contribution statement

**Wenkai Zheng:** Writing – original draft, Investigation, Formal analysis, Data curation. **Wenjin Chen:** Writing – original draft. **Jiao Fang:** Writing – original draft. **Na Li:** Data curation. **Junchao Huang:** Data curation. **Leilei Wang:** Data curation. **Meifang Wang:** Investigation, Data curation. **Xiujuan Feng:** Investigation, Data curation. **Chunni Heng:** Data curation. **Yunlong Tan:** Writing – review & editing, Supervision, Funding acquisition.

## Declaration of competing interest

The authors declare no conflicts of interest.
